# Validation of the North American Chest Pain Rule in Prediction of Very Low-Risk Chest Pain; a Diagnostic Accuracy Study

**Published:** 2017-01-09

**Authors:** Somayeh Valadkhani, Mohammad Jalili, Elham Hesari, Hadi Mirfazaelian

**Affiliations:** 1 Emergency Medicine Department, Kermanshah University of Medical Sciences, Kermanshah, Iran.; 2 Emergency Medicine Department, Tehran University of Medical Sciences, Tehran, Iran.

**Keywords:** Acute coronary syndrome, decision support techniques, emergency service, hospital

## Abstract

**Introduction:**

Acute coronary syndrome accounts for more than 15% of the chest pains. Recently, Hess et al. developed North American Chest Pain Rule (NACPR) to identify very low-risk patients who can be safely discharged from emergency department (ED). The present study aimed to validate this rule in EDs of two academic hospitals.

**Methods:**

A prospective diagnostic accuracy study was conducted on consecutive patients 24 years of age and older presenting to the ED with the chief complaint of acute chest pain, during March 2013 to June 2013. Chest pain characteristics, cardiac history, electrocardiogram findings, and cardiac biomarker measurement of patients were collected and screening performance characteristics of NACPR with 95% confidence interval were calculated using SPSS 21.

**Results:**

From 400 eligible patients with completed follow up, 69 (17.25 %) developed myocardial infarction, 121 (30.25%) underwent coronary revascularization, and 4 (2%) died because of cardiac or unidentifiable causes. By using NACPR, 34 (8.50%) of all the patients could be considered very low- risk and discharged after a brief ED assessment. Among these patients, none developed above-mentioned adverse outcomes within 30 days. Sensitivity, specificity, positive prediction value, and negative prediction value of the rule were 100% (95% CI: 87.35 - 100.00), 45.35 (95% CI: 40.19 - 50.61), 14.52 (95% CI: 10.40 – 19.85), and 100 (95% CI: 97.18 - 100.00), respectively.

**Conclusions:**

The present multicenter study showed that NACPR is a good screening tool for early discharge of patients with very low-risk chest pain from ED.

## Introduction

Acute chest pain is one of the most common chief complaints of patients presenting to emergency department (ED) (1). Acute coronary syndrome (ACS) accounts for more than 15% of chest pains and is prone to misdiagnosis and inappropriate discharge (2). According to several studies, this mishap takes place in 4.4% of ACS cases (3). In a recent research, it has been reported that this apparently low percentage has 9.1% fatal outcome over a 30-day period (4). These concerns have made emergency physicians lower their work-up threshold, which may in turn result in overcrowding, increased costs, and false positive test results (5). In order to reduce the risk, several decision rules have been developed to stratify patients with chest pain (2). Recently, Hess *et al. *tried to establish a decision rule for identification of very low-risk patients who can be safely discharged from ED (6-8). This so-called North American Chest Pain rule (NACPR) has been developed by adherence to the recommendations on prediction rules development. They proposed that patients could be discharged if they were under 40 years old with no new ischemic change on the electrocardiogram (ECG), no past history of coronary artery disease (CAD), no typical chest pain, and initial troponin within normal limits. For the patients aged between 40 and 50 years, a second normal troponin level should be available for patients to be considered as dischargeable. Therefore, the present multicenter study was conducted to validate this prediction rule in two academic EDs.

## Methods

***Study design and setting***

A consecutive prospective diagnostic accuracy study was conducted on adult patients who presented to the EDs of Imam Khomeini and Shariati Hospitals, Tehran, Iran, with chest pain as their primary complaint during March 2013 to June 2013. Informed consent was obtained from every patient and the data were kept confidential. The Institutional Review Board of the hospitals and ethic committee of Tehran University of Medical Sciences approved the study protocol. 

***Participants***

All adults older than 24 years with chief complaint of anterior chest pain presenting to ED were enrolled. As in Hess et al. study, patients were excluded if there was ST segment elevation at least in two contiguous leads on the initial ECG, hemodynamic instability (persistent heart rate greater than 100 beats/min or less than 50 beats/min or systolic blood pressure persistently below 90 mmHg), an unreliable clinical history, a chest pain caused by trauma, a documented history of cocaine abuse (in laboratory study or clinically), a non-cardiac terminal illness, pregnancy, previous enrollment within the past 30 days, or inability to receive follow-up by telephone (8). 

Studied hospitals were tertiary-care university-affiliated centers with more than 600 beds each. They have an annual ED visit rate of approximately 40,000 patients. 

All diagnostic tests and therapeutic procedures were performed at the discretion of the attending physician, according to routine ED practice. Patients presenting to the ED with definite signs and symptoms of developing ACS (e.g. ST-elevation or elevated cardiac biomarkers) were consulted with a cardiologist and admitted directly to the coronary care unit afterwards. Other patiens were admitted and observed in the ED. Serial ECGs and cardiac markers were obtained and further decisions were made according to the results. A team of well-trained research assistants worked in a scheduled set of shifts and enrolled eligible patients. 

***Data gathering***

Relevant data (i.e. patient’s age and sex, history of CAD, and chest pain characteristics) were recorded on specific data collection forms by research assistants. As in Hess *et al.* study, past history of CAD was defined as at least 50% coronary stenosis on angiography; demonstrated ECG changes, perfusion defects, or wall motion abnormalities on exercise, pharmacologic, or rest imaging studies; or previous documentation of acute myocardial infarction (8). According to hospital policies, ED physicians ordered both serum creatine kinase myocardial and brain isoenzymes (CK-MB; CK-MB STAT cobas, Roche Diagnostics, Indianapolis, IN) and troponin T (Elecsys Troponin T Assay, Roche Diagnostics) for ACS patients in the initial hours of admission. In this study, troponin level on arrival was used as per the original study (8). In order to follow the study protocol, if the patient was between 41 and 50 years old and the troponin level on arrival was normal, the second troponin level test was reordered 6 hours after onset of symptoms. The patient’s final diagnosis was made based on the results of cardiac biomarkers, ECG changes and angiographic findings and was gathered from patients’ hospital files. On day 30, one of the investigators (EH), who was blind to the patients’ baseline characteristics and their screening results by NACPR, contacted patients and asked about their health status, symptoms recurrence, and any diagnostic evaluation or therapeutic procedure performed after discharge. The results were recorded on a predesigned data sheet.

Assigning patients to one of NACPR groups was done retrospectively and no intervention was performed to implement the rule during admission. All the ECGs were reviewed by two investigators blinded to final outcome (SV, HM), and a third investigator (MJ) resolved disagreement. The definition in Hess et al. study was used to define ECG abnormality (8). By considering all the factors in the history and physical examination, the clinician classified the chest pain syndrome as typical (i.e. of cardiac cause) or atypical (i.e. of non-cardiac cause).

***Outcomes***

Myocardial infarction (ST-elevation and non ST-elevation), coronary revascularization, cardiac death, and no other definite cause found in investigation were considered as 30 days outcomes. Outcomes were defined same as original derivation study of NACPR (8). The mentioned outcomes were measured after comprehensive data assessment by investigators blinded to the patients’ NACPR screening results. 

***NACPR***

According to NACPR, two groups of patients are eligible for early discharge. The first group includes patients younger than 40 years of age with a normal primary ECG, reporting very low-risk chest pain characteristics, and without history of ischemic chest pain. The second group of patients consist of patients 41-50 years of age with normal troponin level 6 hours after the pain onset, in addition to the criteria mentioned for the first group (8).

***Statistical analysis***

Minimum sample size required for the present study was calculated to be 400 cases, considering 100% sensitivity of NACPR (95% CI: 97.1 -100), 20.9% specificity (95% CI: 16.9 – 24.9), 0.06 p value, and 0.048 desired precision (8). Standard descriptive statistics such as means and standard deviations (SDs) for normally distributed continuous data, medians and inter quartile ranges for skewed continuous data, and frequencies with proportions for categorical data were calculated using SPSS version 21 (SPSS Inc., Chicago, IL). Performance of NACPR for identifying the very-low-risk patients in this study was assessed by calculating sensitivity, specificity, positive predictive value, negative predictive value, and positive and negative likelihood ratios using the statistical software MedCalc® Version 14.10.2, available online at http://www.medcalc.org/calc/diagnostic_test.php.

## Results

***Enrollment***

**Figure 1:**
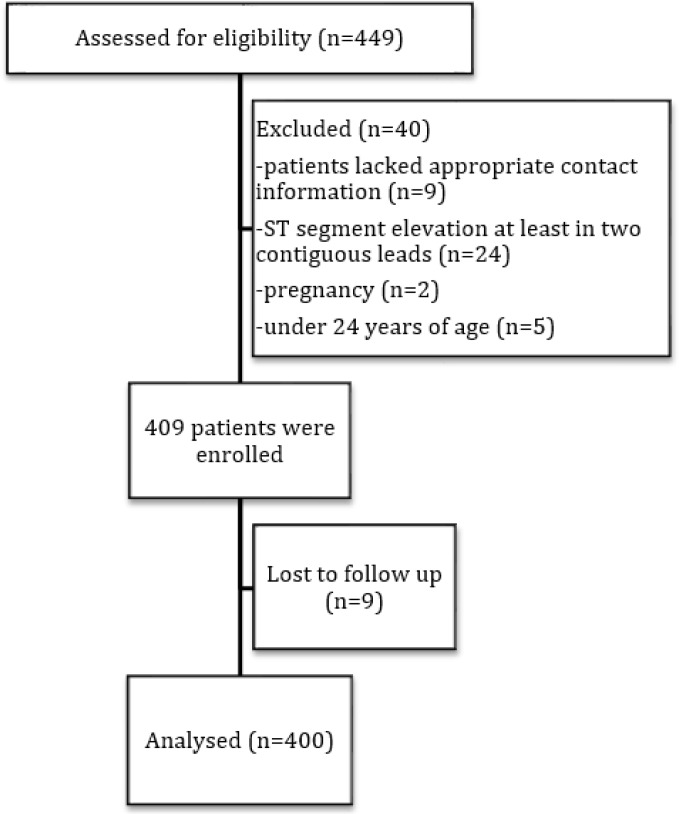
The study flowchart

During the 4-month study period, a total of 449 patients were potentially eligible for enrollment in this study. After screening assessments, 40 of them were excluded (9 patients lacked appropriate contact information, 24 had ST segment elevation at least in two contiguous leads, two were pregnant, and five patients were under 24 years of age). Therefore, a total of 409 patients were finally enrolled. 400 of which had follow-up completed (Figure 1). Reviewing the patient records in triage revealed that 30 patients with chest pain had been missed by research assists.

***Baseline characteristic of patients***

The mean age of the 400 enrolled patients was 56.77 ± 12.52 (25 - 87) and 213 (53.3%) cases were male. The baseline characteristics of patients are summarized in Table 1. Most of the patients were in 40 – 55 age group (40.5%). Hypertension was the most common risk factor (45.5%) and 169 (42.3%) patients had abnormal ECG. 264 (66%) cases of chest pain were typical. Patient dispositions were as follows: 23 (5.8%) cases were discharged from ED, 359 (89.8%) cases were admitted to coronary care unit (CCU), and 18 (4.5%) patients left ED against medical advice. 

***Screening characteristics of rule***

**Figure 2:**
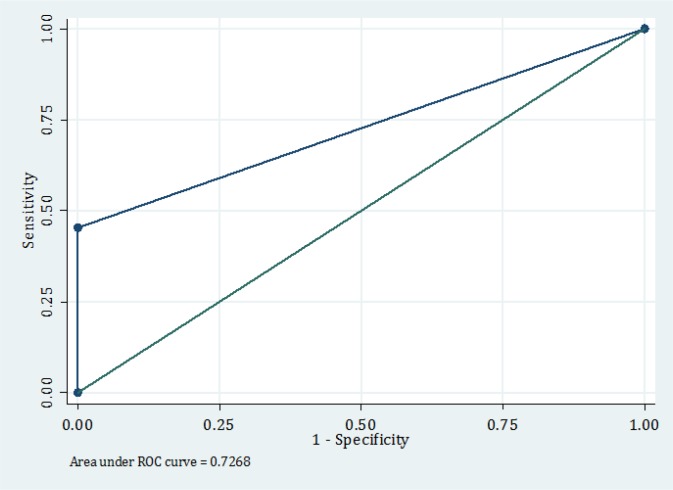
Receiver operating characteristics (ROC) curve of North American Chest Pain rule.

Based on the results of screening with NACPR, 34 (8.5%) cases were in very low-risk group for developing 30 day adverse outcomes and were eligible to be discharged from ED. Table 2 summarizes 30 day adverse outcomes of studied patients. 194 (48.5%) of them had experienced adverse outcomes. Table 3 shows the screening performance characteristics of the prediction rule. The area under the ROC curve was 0.726 (0.681 – 0.770), figure 2. Excluding the second troponin level test in 40-50 year old patients did not change the rule’s performance.

## Discussion

Based on the results of the present study, NACPR has 100% sensitivity and negative predictive value in predicting very low-risk patient for developing 30 day adverse outcomes of MI, revascularization, and death among those presenting to ED with chest pain. Having used NACPR, 34 patients (8.50%) would have been eligible to be included in the very low-risk group and could be discharged from the ED. None of the 166 patients who developed aforementioned outcomes within 30 days would have been included in the very-low-risk group by implementation of this rule. 

In our study, sensitivity and negative likelihood ratio were similar to those found by Hess et al. and Mahler et al. (100% and 0%). The specificity in our study (14.53%) was lower than the original study (20.90%) but higher than Mehler et al. study (5.6%) (8, 9). This difference may be due to the type of patients and the center where our patients were selected. Interestingly, all the patients in 40-50 with abnormal second troponin had another criterion of the rule that excluded them from early ED discharge. As a result, the rule performance would not change after excluding the second troponin measurement.

**Table 1:** Baseline characteristics of studied patients (n = 400)

**Characteristic**	**Number (%)**
**Age (year)**	
25 – 39.9	25 (6.3)
40 – 54.9	162 (40.5)
55 – 69.9	147 (36.8)
≥ 70	66 (16.5)
**Sex **	
Male	217 (53.3)
Female	187 (46.7)
**Medical History **	
Diabetes mellitus	97 (24.3)
Hypertension	183 (45.8)
Hyperlipidemia	112 (28)
Coronary artery disease	176 (44)
Smoking	106 (26.5)
Family history of CAD	8 (2)
**Abnormal ECG**	
ST segment change	78 (19.5)
T wave inversion	169 (42.3)
Total	169 (42.3)
**Troponin enzyme level**	
Normal	299 (74.8)
Abnormal	101 (25.3)
**Type of chest pain**	
Typical	264 (66)
Atypical	136 (34)

CAD: coronary artery disease; ECG: electrocardiogram.

**Table 2:** 30 day outcomes of studied patients

**Outcomes**	**N (%)**
**Myocardial infarction**	
ST segment elevation	15 (3.8)
Non- ST segment elevation	54 (13.5)
**Revascularization**	
Percutaneous coronary intervention	86 (21.5)
Coronary artery bypass graft	35 (8.8)
**Survival**	
Dead	4 (1)
Alive	396 (99)

**Table 3:** Screening performance characteristics of North American Chest Pain rule in prediction of very-low-risk patient with chest pain

**Characteristics**	**Value (95% confidence interval)**
**Sensitivity **	100 (87.35 - 100.00)
**Specificity **	45.35 (40.19 - 50.61)
**Positive predictive value**	14.52 (10.40 – 19.85)
**Negative predictive value **	100 (97.18 - 100.00)
**Positive likelihood ratio **	0.17 (0.12 to 0.23)
**Negative likelihood ratio**	0
**Accuracy**	0.726 (0.681 – 770)

Clinical decision rules are developed in order to facilitate the decision making process in common and important clinical conditions. Acute chest pain has a high prevalence, which calls attention to develop rules. These rules use easily obtainable elements in history, clinical examination, and simple tests, to safely rule out hazardous conditions (2). 

Data in the United States showed that approximately 2% of patients with ACS are misdiagnosed and inappropriately discharged from the EDs (3). Furthermore, several patients suspected of having ACS are ultimately diagnosed with non-cardiac chest pain and sent home after time-consuming and costly workups (10). This sheds light on the necessity of a set of screening criteria with high sensitivity, to minimize misrate, and with high specificity, to prevent excessive costs arising from diagnostic procedures and long hospital stays. 

In 1990s, some rules for cardiac care unit admission were developed based on observation with 3% misrate (11, 12). Later on, biomarkers were employed to develop rules (e.g. TIMI risk score) and in order to improve this process, several standards were defined. In this regard, according to systematic reviews, many of chest pain decision rule derivation studies are retrospective with methodologic flaws (13, 14). 

In a prospective study, Hess et al. derived a clinical decision rule for identification of patients with very low-risk chest pain who could be safely discharged from ED without further objective cardiac testing such as stress tests (8). By adhering to the correct methodology, the researchers followed 2718 patients for 30 days. 336 patients experienced adverse cardiac events. By analyzing the data, they proposed that patients could be discharged if they were under 40 with no new ischemic change present on the ECG, no past history of CAD, without typical chest pain, and initial troponin within normal limits. For the patients between 40 and 50, a second normal troponin level should be available after 6 hour of symptom onset for patients to be considered as dischargeable.

Recently Vancouver Chest Pain Rule was developed according to methodological standards by utilizing biomarkers (15). Christenson et al. used past history and initial ECG to discharge patients younger than 40. For older patients they proposed using pain characteristics and CK-MB level. This study was 98.8% sensitive and 32.5% specific for prediction of adverse cardiac events within 30 days. 

Jalili et al. found sensitivity of 95.1% and specificity of 56.3% for Vancouver Chest Pain Rule. The PPV and NPV were fairly similar (2). 

In addition to having lower yields in comparison to NACPR, there are some debates over this rule. For example, Hess et al. study showed that some elements in Vancouver Chest Pain Rule have insufficient inter-observer reliability (8). Recently, Mahler et al. compared the NACPR with HEART score for major cardiac events and unstructured clinical evaluation (9, 16). Since the study was a secondary analysis on another study, definition of elements of the rule (i.e. ECG interpretation, chest pain description, past history of CAD, and serial troponin timing (0 and 3 hours)) differed from Hess et al. study. Interestingly, the sensitivity and negative likelihood ratio of this modified NACPR was 100% and zero, respectively. The results showed that both rules were comparable with unstructured clinical evaluation and had the acceptable misrate of less than 1%.

***Limitations***

Our study faces some limitations. Although this rule assesses chest pain, like Hess et al. study, we did not study unstable angina independently. In addition, non-chest pain presentation of cardiac origin was not included in the rule, which precludes its application in these patients. During the study period, inter-observer agreement was not assessed in our study in regard to gathering patient information by research assistants and ECG assessment by HM and SV, which in turn may increases the risk of performance bias. As shown in the Hess et al. study, several other factors such as presence or absence of observation units can also affect the costs. However, due to lack of accurate and detailed financial records, we were not able to determine the use of financial resources and find out if costs had been minimized by early discharge of very-low-risk patients. Another limitation in our result interpretation is 10% failed enrollment or follow up. In addition, even the most accurate rules are unlikely to be applied in practice if they are not considered reasonable by the end-user, therefore, these rules should consist of simple and sensible elements (17). End-user (physician) contentment was not assessed in our study either. 

## Conclusion:

In summary, our study proved that the prediction rule proposed by Hess et al. is a sensitive decision tool for diagnosing very-low-risk chest pain patients who can be discharged early from the ED.
